# Trends in Malaria Cases and Deaths: Assessing National Prevention and Control Progress in Burundi

**DOI:** 10.24248/eahrj.v4i2.642

**Published:** 2020-11-26

**Authors:** Adolphe Ndoreraho, Muhammed Shakir, Celestine Ameh, Chukwuma Umeokonkwo, Olusola Aruna, Juma Ndereye, Ayo Adebowale

**Affiliations:** a Muramvya Provincial Health Bureau, Burundi Ministry of Health and fight against AIDS; b Nigeria Field Epidemiology and Laboratory Training Program, Abuja; c Public Health England/Nigeria Centre for Disease Control; d Ministère de la SantéPublique et de la Luttecontre le Sida du Burundi; e Department of Epidemiology and Medical Statistics, Faculty of Public Health, College of Medicine, University of Ibadan, Ibadan

## Abstract

**Background:**

Malaria is associated with high morbidity and mortality especially in World's tropical regions. In 2016, an estimated 216 million and 445,000 cases of malaria and deaths associated with malaria respectively were reported globally. Malaria is the first leading cause of outpatient visits, hospitalization and death in Burundi. We therefore examined the trend in malaria cases and deaths in Burundi.

**Methods:**

We extracted data from Burundi National Health Information System (BNHIS) and assessed trends in malaria cases and deaths from January 2015 to December 2017. A suspected case of malaria was defined as any person treated by anti-malarial drugs without testing while a confirmed case as any person with a positive microscopy or rapid diagnostic test for malaria parasite. We described malaria cases and deaths, and calculated malaria case incidence rate.

**Results:**

A total of22,225,699 malaria cases with 8,660 deaths (CFR 0.04%) was documented during the study period. Out of 22,225,699 cases, 45,291 cases (0.2%) were suspected malaria cases. The observed peak season of malaria infection in any of the studied year was in the raining season (March-June). All provinces of the country were affected. Kirundo and Cankuzo provinces the incidence of malaria cases increased from 10.1 cases per 1,000 persons in 2015 to 13.2 cases per 1,000 persons in 2017. The case fatality rate decreased from 0.06% in 2015 to 0.01% in 2017.

**Conclusion:**

An increasing trend in malaria prevalence was observed in Burundi but Kirundo and Cankuzo provinces were the most affected. However, the case fatality decreased within the studied period. Malaria intervention should be intensified/scaled up in the raining season and the most affected provinces.

## BACKGROUND

Malaria remains a threat to public health, particularly in sub-Saharan Africa, which is home to 90% of the world's malaria cases.^1^ In 2016, this African sub-region had about 64,000 malaria deaths and 38 million reported cases, of which 32 million were confirmed malaria cases. The Democratic Republic of the Congo accounted for 48% of these reported cases, followed by Burundi (26%) and Angola (12%). Nine countries had increased cases from 2015 to2016. Angola and Burundi alone reported 3.8 and 8.3 million confirmed cases in 2016, which represents a 60% and 37% increase from 2015, respectively.^2^ Due to high mortality associated with malaria in Burundi and some countries in sub-Saharan Africa, one of the Sustainable Development Goals (SDGs) targets of reducing child mortality might be unrealizable if the trend continues.

Malaria is the main cause of mortality in pregnant women and children below five years of age^3^ and continues to kill millions of Burundians, despite concerted efforts to reduce malaria mortality. This is often attributed to a number of factors, including poor immunological competence because of malnutrition, climate change responsible for rising temperatures and increased rainfall and poverty, limited access to basic health care and specialized health facilities.^4^ Three species of Plasmodium are present and affect the population differently. Plasmodium falciparum is the most formidable species because it is responsible for serious fatal forms and according to existing data, it is the cause of more than 90% of the infections encountered in Burundi. The other two species (*Plasmodium falciparum and Plasmodium ovale*) represent only 8% and 2% respectively. Mixed infections with *P. falciparum* and *P. ovale* also exist.^5^

Burundi's Strategic Plan for the fight against malaria covers interventions aimed at reducing malaria; including universal Insecticide Treated Bed Net (ITN). The plan also includes strategies to improve malaria case management, improve diagnostic testing capacity and quality, increase coverage of three doses of sulfadoxinepyrimethamine (SP) for intermittent preventive treatment in pregnancy (IPTp), establish a robust surveillance system, and establish a monitoring and evaluation framework.^3^

In Burundi, the results of the evaluation of routine distribution of long-lasting insecticidal nets (LLINs) show that coverage is higher in urban than in rural areas. Indeed, 75% of urban households versus 62% of rural households own at least one ITN or LLIN. The average number of ITNs or LLINs is estimated at 1.5 per household in urban areas compared to 1.1 in rural areas.^6^ In Burundi, the results of the evaluation of routine distribution of LLINs show that coverage is higher in urban than in rural areas. Indeed, 75% of urban households versus 62% of rural households own at least one ITN or LLIN. The average number of ITNs or LLINs is estimated at 1.5 per household in urban areas compared to 1.1 in rural areas.^6^

Understanding malaria cases and deaths trends is critical for evaluation of progress made or assessing gaps in the malaria control efforts in Burundi. Reducing the incidence of malaria cases and deaths is a national priority that requires a focused, comprehensive, and consistent approach in order to achieve the vision of “a malaria-free by 2030”, as stated in the 2016-2017 Burundi National health policy (PNS).^7^ We described the national and provincial malaria cases and deaths from 2015 to 2017, highlighting vulnerable populations, and comparing proportions of laboratory-confirmed malaria cases reported from Burundi's eighteen provinces. Our objective was to assess the trends in malaria cases and deaths in Burundi from 2015 to 2017.

## METHODS

### Study Setting

Burundi is located in the eastern part of Africa and is divided administratively into 18 provinces. The 2017 projected population was estimated to be 9,978,423 with 50.8% women and an annual population growth of 2.4%. Children under 5 years of age represent 19.3% of the total population. Burundi covers an area of square kilometre 27,834.^8^ Its climate is tropical with four seasons, a small rainy season (October to December), a small dry season (January to February), the great rainy season (March to May) and the great dry season (June to September).^7^ The country's healthcare coordination is organized into three pyramidal and hierarchical levels: the central, intermediate and peripheral levels. The central level consists of the office of the minister, a general health inspectorate, two general directorates, 6 departments, 9 health programs and related services. The intermediate level comprises 18 provincial health bureaux^9^. The peripheral level has 46 health districts, 91 hospitals and 1,057 primary health-care centres.^10^

Malaria is one of the nationally diseases under surveil-lance reported on a both weekly and monthly basis. However, during epidemics the reporting is changed to daily form. Public health facilities, private and No Governmental Organizations (NGO) health facilities are the reporting entities. Clinical staff records health data. This data is then sent weekly and monthly to the Districts data managers. District data managers then compile reports from health facilities and send them to provincial data managers, which in turn, submit this information to the central level. The collected malaria data is reported from health facilities through either a focal person's email or mobile texting.^11^ The population under surveillance for malaria is the total population of the country.

### Study Design and Data Source

This is a secondary data analysis of routinely collected countrywide malaria data from both hospitals and health facilities in the 18 provinces in Burundi. We obtained data from Burundi National Health Information System (BNHIS) for the period from 2015 to 2017.

Permission to analyze the data was obtained from Burundi Ministry of Health. We extracted provincial data on all malaria cases reported in the District Health Information Software 2 (*DHIS2*) from 2015 to 2017. Variables extracted from the surveillance system included total reported malaria cases and laboratory confirmed malaria cases. A suspected malaria case was defined as any person treated by anti-malarial drugs without testing. A confirmed case was a person who tested positive for malaria parasite. The extracted data was exported into an Excel 2016 workbook. All the characteristics at inclusion, as well as the variables of interest were described in terms of numbers and percentages from the cross-tabulations and frequency tables. The QGIS 2.18.13 software allowed us to produce the flat maps of our study area.

### Data Analysis

Malaria prevalence and specific mortality rates were calculated using the mid-year population, which was estimated from the projected population based on the 2008 census and assuming a 2.4% annual population growth each year. The prevalence of malaria was estimated per year. The malaria mortality rate was defined as the number of deaths among confirmed malaria cases divided by the mid-year population of the district, while prevalence was defined as the number of reported malaria cases divided by the mid-year population. Charts and frequency were used to describe the trend.

### Ethical Consideration

This is a secondary data analysis without personal identification information, not human subject research. Permission to use the malaria morbidity data was obtained from and grated by Burundi National Health Information System of the Ministry of Health. All data extracted were confidentially stored at the end of the study.

## RESULTS

**FIGURE 1.**
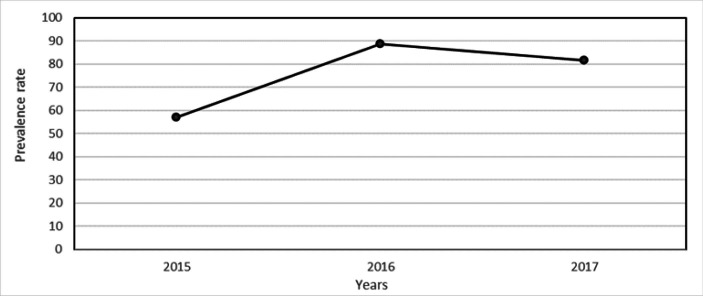
Annual Prevalence Rates of Total Malaria Cases (All) Per 100 Populations 2015-2017

A total of 22,225,699 malaria cases were reported from January 2015 to December, 2017, and almost all cases (99.8%) were parasitological confirmed. The malaria contribution to the outpatient cases was 41.5% with less proportion of malaria cases being recorded in 2015 (Table 1). Malaria prevalence was highest in 2016 with 57.0/100 persons (Figure 1). The Case Fatality Rate (CFR) decreased from 0.06% in 2015 to 0.01% in 2017 (Figure 3). The decrease in CFR was not statistically significant (*p=.21*).

**TABLE 1:** Outpatient Malaria Cases Reported in Burundi from 2015 To 2017

Year	Outpatient all causes	TotalMalaria Cases	%	Confrmed cases	%
2015	16,138,956	5,426,400	33.6	5,408,809	99.7
2016	19,368,945	8,658,050	44.7	8,637,680	99.8
2017	18,099,575	8,141,249	45.0	8,133,919	99.9
**Total**	**53,607,476**	**22,225,699**	**41.5**	**22,180,408**	**99.8**

**FIGURE 2.**
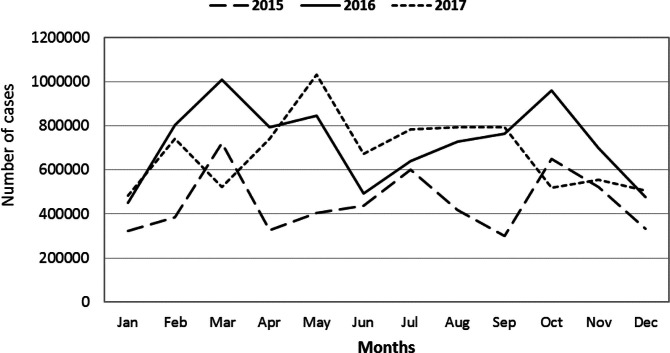
Seasonal Indexes of 3 Years Malaria Cases in Burundi, 2015-2017

**FIGURE 3.**
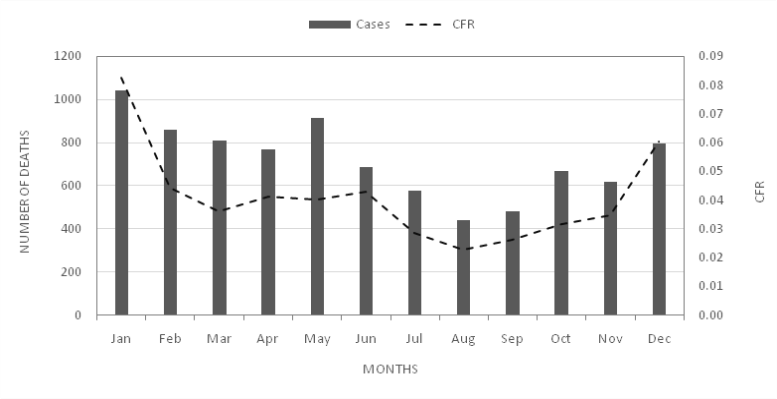
Compilated Malaria Deaths/CFR per Month in Burundi 2015-2017

The highest number of malaria cases was recorded in 201-6 (8,658,050 cases), while the lowest was recorded in 2015 (5,426,400 cases). The highest standard deviation was observed in 2016, meaning that cases reported in that year had the highest monthly variation, while the lowest standard deviation was recorded in 2015, meaning that monthly reported cases did not vary significantly (Table 2).

**TABLE 2.** Mean Number of Malaria Cases in Burundi, From 2015 To 2017

Year	Mean	Standard deviation	Minimum	Maximum	Total
2015	452,200	140,266.4	299,776	722,790	5,426,400
2016	721,504.2	181,442.1	450,988	1,010,098	8,658,050
2017	678,437.4	166,122.1	482,710	1,030,712	8,141,249

Malaria occurred every month and the distribution of cases per month showed a monthly and yearly fluctuation in trend in the years under study. Cases peaked in March of 2015 and 2016 and in May of 2017 (Figure 3). In addition, the result of Chi-square analysis revealed statistically significant association of malaria cases and seasons (x^2^ = 713661.41, df = 22, *p <.0001*).

**FIGURE 4.**
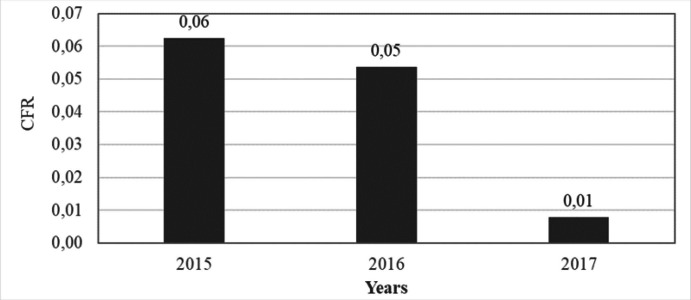
Malaria Case Fatality Rate Trends in Burundi, 2015-2017

Kirundo and Cankuzo provinces had the highest changes in incidence, increasing from 101 cases per 1,000 persons in 2015 to 137 cases per 1,000 persons in 2017 for Cankuzo province and 113 cases per 1,000 persons in 2015 to 135 cases per 1,000 persons in 2017 for Kirundo province. (Figure 4).

**FIGURE 5.**
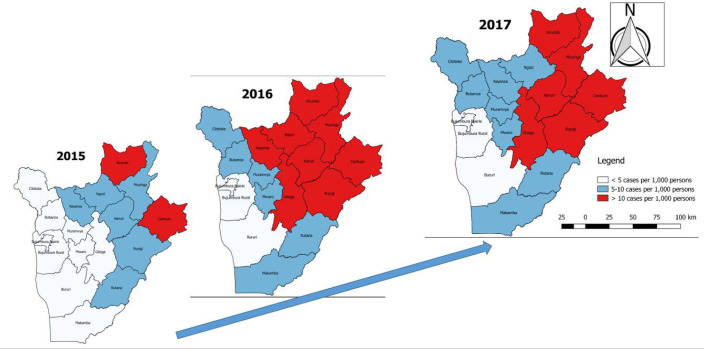
Malaria Prevalence Trends by Province in Burundi 2015-2017

## DISCUSSION

The present study demonstrated that malaria incidence fluctuated annually, with the minimum and maximum number of cases recorded in 2015 and 2016, respectively. The maximum number of malaria cases recorded in 2016 despite the mass distribution campaign of long-lasting insecticide treated mosquito nets in 2014 may be attributed to community misconception surrounding malaria prevention and control. Moreover, the ITNs alone is not enough to control malaria. Its combination with other strategies including vector control, pharmacovigilance of ant malarial drugs, etc.

The effectiveness of the national response strategy undertaken to control malaria in the year 2016 might be the reason for the decrease of the prevalence to 81.5 % in 2017. The strategy includes universal insecticide-treated bed net (ITN), malaria case management, improve diagnostic testing capacity and quality, increase coverage of three doses of *sulfadoxinepyrimethamine* (SP) for intermittent preventive treatment in pregnancy (IPTp), establish a robust surveillance system, and establish a monitoring and evaluation framework. However, this finding still higher than that of the Burundi Demographic and Health Survey (BDHS) which revealed a prevalence of 51%^12^. This may be due the fact that our study used the country wide data obtained from the surveillance system while the BDHS used a sample since there is the possibility of sampling bias in favour of those who did not have malaria two weeks before the survey as defined in BDHS protocol.

The percentage of confirmed cases among those who reported that they show symptoms of malaria ranged from 99.7 % in 2015 to 99.9 % in 2017. High rate of confirmed cases among the reported cases found in this study is an indication of good knowledge and correct diagnosis of malaria by the Burundians without the use laboratory confirmation. An increasing proportion of reported malaria cases with laboratory confirmation can signify improved adherence to diagnostic and treatment guidelines for malaria and stronger surveillance.

However, the finding from this present study with respect to high percentage of confirmed cases among reported cases is greater than the finding from the 2015 survey on the availability, accessibility and use of malaria control inputs as well as the quality of malaria care in selected health facilities, where the proportion of confirmed malaria cases in all fever cases (confirmation rate) was 72%.^13^

The difference could be attributed to the fact that 2015 survey on the availability, accessibility and use of malaria control inputs as well as the quality of malaria care in selected health facilities was conducted in some selected heath centres in Burundi and not population based as observed in the surveillance data which more enhanced and robust than the health facility survey.

In this study, malaria case fatality rate showed a decreasing trend from 2015 to 2017 (0.06% to 0.01%). This should be attributable to the scaling-up of RDT based rapid case detection contributing especially during the malaria outbreak of 2017. A study conducted in India where malarial mortality was showing gradual decrease in trend corroborate our finding.^14^

Malaria cases occur in all month and years in this study area. The highest peak malaria case was observed in March, May and October. This may be the consequence of the Burundian rainy seasons which runs from March to October of a particular year create a favourable conditions and environment for the breading of mosquitoes. Kirundo and Cankuzo provinces consistently had the highest incidence of malaria cases from 10.1 cases per 1,000 persons in 2015 to 13.2 cases per 1,000 persons in 2017. This pattern is similar to the findings from the Burundian malaria indicators survey of 2012.^15^ Reducing the incidence of malaria is a national priority that requires a focused, comprehensive, and consistent approach in order to achieve the vision of “a malaria-free by 2030”, as stated in the 2016-2017 Burundi National health policy (PNS).^7^

## CONCLUSION

Malaria occurred in all months throughout the years under investigation and all provinces in Burundi were affected by malaria but substantial variation was expressed across the province. Laboratory confirmation of malaria in the 3-year period under review was on the increase. However, cases peaked in March, May and October. The case fatality rate gradually declined. Therefore, the existing malaria prevention and control programs should be scaled-up and strengthened in Burundi. Appropriate disease and vector control strategies must be implemented.

### Limitations

Individual-level data were not available and consequently data on age or sex distribution were not provided. This limited our ability to determine malaria prevalence by sex as well as other potential risk factors. Lack of availability of station level climate data, more detailed role of climatic conditions on the spatial patterns of malaria prevalence could not be assessed.

### Authors’ Contributions

Adolphe Ndoreraho was responsible for data sorting, data analysis, manuscript drafting. Muhammed Shakir and Ayo Adebowale provided technical support in terms of data analyses and reporting. JumaNdereye, Olusola Aruna, Chukwuma Umeokonkwo and Celestine Ameh contributed to the writing of the introduction and discussion section of the paper. All the authors reviewed and approved the final version of the manuscript.
